# Hot Topics in Cellular Neuropathology II: Promoting Neuronal Plasticity in the Injured Central Nervous System

**DOI:** 10.3389/fncel.2022.927980

**Published:** 2022-06-03

**Authors:** Dirk M. Hermann

**Affiliations:** Department of Neurology, University Hospital Essen, University of Duisburg-Essen, Duisburg, Germany

**Keywords:** neuroplasticity, axonal plasticity, dendritic plasticity, synaptic plasticity, functional neurological recovery, excellence, innovation, therapy

Progress in Cellular Neuropathology is strongly facilitated by paradigm changes. Paradigm changes shift the way we look at neurological diseases. They promote new experimental approaches, from which translational advances in clinical neurosciences may result. Clinical neurosciences were predominated for ~80 years by the doctrine of the Spanish neuropathologist Santiago Ramon y Cajal (1852–1934), the discoverer of the axonal growth cone. In meticulous neuropathological studies, Ramon y Cajal has made fundamental contributions to clinical neurosciences by pursuing the concept of neurons as contiguous entities. Photographs of his silver nitrate stainings, which he advanced based on Camillo Golgi's (1843–1926) protocols, are legendary. Besides others, Ramon y Cajal postulated that adult central nervous system (CNS) neurons are able to sprout and create new axonal growth cone connections, which he considered as structural correlate of memory formation and learning (Sherrington, 1935). In response to CNS injury on the contrary, using autopsies of patients who died post-CNS injury, Ramon y Cajal observed surprisingly little axonal growth across and beyond brain or spinal cord lesions. Ramon y Cajal concluded that CNS axons of adult human neurons exhibit limited potential for white matter tract regeneration and sprouting (Ramon y Cajal, 1928). This neuroregeneration failure he made responsible for deficient neurological recovery in CNS injured patients.

Following a long interval, in which neuroregeneration in the adult CNS was considered futile and not further pursued, experimental studies in the 1980ies and 1990ies demonstrated that the limited neuronal plasticity in the adult mammalian CNS is a consequence of axonal growth inhibitors released by oligodendrocytes and astroglia, which actively prevent neuronal sprouting (Schwab, 1990). These growth inhibitors, which are proteins or proteoglycans, are downregulated in response to CNS injury, enabling restricted axonal outgrowth associated with spontaneous neurological improvements (Schwab, 1990). Interestingly, the neutralization of axonal growth inhibitors by delivery of neutralizing antibodies directed against these growth inhibitors rigorously increased axonal sprouting *in vitro* and *in vivo* and enhanced functional neurological recovery in spinal cord injury models (Schnell and Schwab, 1990; GrandPré et al., 2002) and more recently also in ischemic stroke models (Clarkson et al., 2010; Reitmeir et al., 2011; Wahl et al., 2014). Currently, considerable efforts are made to establish neurorestorative treatments following CNS injury. First clinical proof-of-concept studies using strategies that promote neuronal plasticity made promising observations (Chollet et al., 2011) and demonstrated the feasibility of plasticity-promoting treatment studies. Subsequent studies identified a number of methodological pitfalls related to the selection of therapeutic targets, the use of clinical endpoints and scales, and the size of study cohorts (FOCUS Trial Collaboration, 2019; Chabriat et al., 2020; Hermann et al., 2020). With the use of adequately designed and powered studies, chances appear favorable that plasticity-promoting treatments can be translated successfully into clinics.

In an effort to identify the most promising concepts in translational neurosciences, the Cellular Neuropathology section of Frontiers in Cellular Neurosciences recently launched a new platform, the Hot Topics Hub (Hermann, 2020). Within this platform, this journal searches for impactful papers in the Cellular Neuropathology field, which carry landscape-changing potential, broaden imagination horizons and expand current diagnostic or therapeutic possibilities. Within this platform, a first very successful series of papers evaluated the role of subtle neuroinflammation in chronic neurodegeneration (Hermann et al., 2022). The papers published within this series examined how subtle, unbalanced neuroinflammation persisting in the chronic phase of brain injury unfavorably influences brain integrity and function, similar to the subtle neuroinflammation associated with psychosocial stress or major depressive disorder (Hermann et al., 2022). Due to the persistent neuroinflammation, brain injury, psychosocial stress and major depressive disorder may result in long-term physical, cognitive, and mental health deficits. The similarities of pathophysiological processes in these diverse settings are intriguing, raising the question, whether the resulting health deficits may be improved by treatments that rebalance CNS inflammatory processes. As a pathophysiological element that connects these diverse conditions, microglial overactivation associated with oxidative stress, cytokine release and disturbed neuronal plasticity has been identified.

Within this new Research Topic, we would like to shift the focus from degenerative to restorative processes, searching papers analyzing the role of neuronal plasticity and the effect of plasticity-promoting treatments in the injured CNS. As in the previous Research Topic, suitable manuscripts should push our understanding of neurological diseases, overcome existing limitations, pave the way for therapeutic progress and deserve attention in future research developments. Papers on axonal, dendritic or synaptic plasticity are welcome using *in vitro* or *in vivo* models of CNS trauma, ischemia, slowly evolving neurodegeneration, infection or cancer, besides others. Papers submitted may further scrutinize molecular mechanisms underlying axonal, dendritic or synaptic plasticity *in vitro* or *in vivo*. *In vitro* or *in vivo* studies may also examine how cellular interactions of neurons, e.g., with astrocytes or microglia, modify plasticity processes. There is a major need for such studies, since these studies may allow us to identify targets for plasticity-promoting therapies. *In vivo* studies might evaluate structural correlates of neuronal plasticity using innovative neuroimaging tools, such as 2-photon microscopy, light sheet microscopy or superresolution microscopy, which provide information about neuronal plasticity in real time in live animals and in 3D. *In vivo* or *ex vivo* studies may link functional changes on the single cell or network level to clinical neurological recovery. The evaluation of neurological recovery in animals following CNS injury is a demanding task, and there is still a need for refined sensorimotor and cognitive tests that reliably detect clinically relevant deficits. In this search for the best ideas and concepts, Original research, Reviews, Perspectives and Opinions are welcome. Papers will be reviewed based on excellence, originality and innovation potential. Outstanding papers will be featured in an editorial. We are curiously looking forward to your contributions to this new Research Topic within the Hot Topics Hub.

## Author Contributions

DMH drafted the paper and revised it.

## Conflict of Interest

The author declares that the research was conducted in the absence of any commercial or financial relationships that could be construed as a potential conflict of interest.

## Publisher's Note

All claims expressed in this article are solely those of the authors and do not necessarily represent those of their affiliated organizations, or those of the publisher, the editors and the reviewers. Any product that may be evaluated in this article, or claim that may be made by its manufacturer, is not guaranteed or endorsed by the publisher.
